# Supplementary material for: Are carbon emissions trading and green financial instruments synergistic? -Comprehensive quantitative research based on content analysis
Source: PLoS One. 2024 Mar 7;19(3):e0298601. doi: 10.1371/journal.pone.0298601 (PMC10919601; doi:10.1371/journal.pone.0298601)
Supplement: S1 Data — (ZIP) [file pone.0298601.s001.zip › Data/地级市Z_F工作报告.zip!/╜¡╦╒/╤∩╓▌╩╨/└┤╫╘╣1⁄2╓┌║┼í╛┬φ┐╦╩2╛▌═°í┐_2012.docx]

各位代表：

现在，我代表扬州市人民政府向大会作工作报告，请予审议，并请市政协各位委员和其他列席人员提出意见。

2011年政府工作回顾

2011年是“十二五”开局之年。面对复杂的国际形势和国内宏观调控的新情况，我们在中共扬州市委的正确领导下，团结和依靠全市人民，紧紧围绕建设“三个扬州”目标，解放思想、克难求进、开拓创新，较好地完成了市六届人大四次会议确定的各项任务。预计全市实现地区生产总值2630亿元，增长12%。财政总收入500.96亿元，增长25%；一般预算收入218.08亿元，增长30%。城市居民人均可支配收入24700元，增长13.5%；农民人均纯收入11200元，增长18.5%。全市总体上全面建成小康社会。

一、经济保持平稳较快增长

工业经济质态提高。全市规模以上工业实现利税770亿元，增长40%。仪化产值突破200亿元；新增中海、晶澳、环洲产值过百亿元企业3家，总数达到6家；新增产值过50亿元企业2家，总数达到9家。新开工10亿元以上项目40项，在建40亿元以上项目4项，上海大众整车、中材LED外延片等一批重大项目顺利推进，亚星与潍柴重组加快实施，扬农集团与中化国际合作正式签约。亚威股份和仁恒实业成功上市，宝胜股份顺利增发。建筑业获鲁班奖4项、国优工程7项，创历史最好水平。

服务业加快发展。制定出台《关于进一步加快发展现代服务业的若干意见》、加快发展软件与信息服务业等9个重点产业提速行动计划和5个专项扶持政策。交通银行金融服务中心落户扬州。新宇软件、国药医药物流、江苏（扬州）影视制作外包基地等项目投入运营。邗江文化创意产业园、江都惠普软件园、仪征物联网产业园等载体建设初具规模。来扬游客和旅游业总收入均增长20%。茱萸湾创成国家4A级景区。浦发银行扬州分行、光大银行扬州分行挂牌营业。

现代农业稳步发展。出台推进水利现代化建设实施意见。粮食连续8年丰收，稻麦两熟亩产实现吨粮。新增高效农业（渔业）园区157个、面积27.6万亩，省级农业产业园区实现县（市）全覆盖。新建农民专业合作社376个。海峡两岸（扬州）农业合作试验区新增项目560个。农业龙头企业销售增长21.2%。新创农业“三品”品牌473个。农业开发、农业科研、粮食、农机、供销、气象等工作取得新成效。成功承办第十三届江苏农业国际合作洽谈会。

开发开放大力推进。沿江岸线实现全线对外开放。高效开展境内外专业招商推介活动，成功举办“烟花三月”国际经贸旅游节、世界运河名城博览会暨首届全球设计城市峰会。全市注册外资实际到账21亿美元，列全省第6位。新增民营企业注册资本金772亿元，增长22.1%。实现进出口总额101亿美元，增长23%。完成外经营业额3.4亿美元，增长44%。玛切嘉利、日本JFE等项目投产，美国应用材料公司、普莱克斯工业气体公司等5个世界500强企业项目落户。扬州经济技术开发区列全国新升级国家开发区综合排名第一，8个省级以上开发区综合发展水平进入全省第一板块。扬州连续10年被评为“台商大陆投资极力推荐城市”。

二、创新转型步伐加快

科技创新成效显著。完成高新技术产业产值2900亿元，占规模以上工业产值比重42%。全社会研发投入占地区生产总值比重2%。新建国家级质检中心1家。新获批国家高新技术企业74家，省级以上研发机构51家。南大扬州光电研究院入选省重大产学研联合创新载体建设计划。省级以上科技创业园孵化面积累计117.5万平方米，在孵企业917家。新引进国家“千人计划”人才7人、高层次领军人才229人。新增“中国驰名商标”7件、省名牌产品28个、专利申请数1.3万件。扬州和高邮、江都、邗江分别被评为全国科技进步先进市和先进县（区）。

新兴产业持续发展。“三新一网一书”实现产值1640亿元，增长45%。保利协鑫、国电南自等行业领军企业项目落户。康博多晶硅、亿霖电子、德豪润达、汇成微电子等项目有序推进。扬州成为国家数字出版基地。

投资消费保持增长。全社会固定资产投资1515亿元，增长25%。投资结构进一步优化，服务业投资530亿元，增长31%。市区房地产开发投资108亿元，增长21%。全市社会消费品零售总额846.1亿元，增长17.6%。

节能减排扎实开展。万元地区生产总值能耗下降3.3%，化学需氧量、氨氮、二氧化硫、氮氧化物排放量分别削减2.5%、2.0%、2.5%和2.8%，完成节能减排年度目标。

三、城乡建设再上台阶

重大基础设施建设加快推进。扬州泰州机场航站楼及附属设施全部封顶。安大公路全线通车。沪陕高速江六段建设进展顺利。宁启铁路复线及电气化改造有序推进。淮河入江水道治理工程正式开工。施桥、邵伯三线船闸改造竣工通航。乌塔沟分洪道加快建设。扬州西500千伏输变电、华电扬州天然气发电等项目稳步推进。连淮扬镇铁路和长江公铁大桥项目上报铁道部。宁扬城际轨道交通列入省“十二五”及中长期发展规划。

中心城市功能进一步完善。顺利实施部分行政区划调整，市区政区结构得到优化，中心城市首位度显著提升。开工建设瘦西湖地下隧道、扬菱路改造等重大工程。文昌路东延及廖家沟大桥、芒稻河大桥建设顺利推进。

甘泉路、广陵路和彩衣街改造基本完成。运博会永久会址建成并投入使用。城庆广场、科技馆、体育中心体育场开工建设。新改建公交站棚50座、公交停车场和首末站5个，建成市区出租车服务中心。制订实施城市内涝整治三年行动方案，整治积水点15处。完成市区第一水厂、六圩污水处理厂一期提标改造工程。“智慧城市”建设全面启动。数字化城管体系进一步完善。

城镇化和新农村建设稳步推进。基本完成11个重点中心镇总体规划和控制性详规编制。县城镇建设步伐加快。66个小城镇建成区面积173平方公里。区域供水江都、高邮实现全覆盖，宝应达到80%。新改建农村公路230公里，新开通镇村公交线路25条。启动实施“美好城乡建设行动”。疏浚县乡河道、村庄河塘3800万方。新建农村无害化卫生户厕7.7万座。强力推进了52个集体经济薄弱村帮扶工作，村级组织“四有一责”建设取得明显成效。

四、民生工作扎实推进

大力推动就业创业。新增城镇就业5.27万人，城镇零就业家庭连续52个月保持动态清零。新增农村劳动力转移4.4万人。开展创业培训1.17万人，新建创业孵化基地18个。

社会保障体系不断完善。出台并实施城乡居民社会养老保险制度，基本实现城乡社会养老保险全覆盖。企业职工基本养老保险新增参保6.3万人、职工基本医疗保险新增参保2.5万人、工伤保险新增参保4.2万人。为全市21万企业退休人员上调养老金并确保发放。城镇居民基本医疗保险参保人数64.65万人。新农保、新农合参保率均达98%以上。实施市区被征地农民和失地农民参加企业职工基本养老保险，已申报4.35万人。邗江、广陵、扬州经济技术开发区实现城乡低保一体化。

民生实事工程较好完成。居民消费价格指数低于全省平均水平。市区新建菜篮子基地2500亩、培植平价蔬菜商店46家、向困难群众发放价格补贴1574万元。“三无”老年居民生活补贴和尊老金发放提标扩面。全市新建廉租房600套、经济适用房1590套、公共租赁房8527套（间）。市区公房解危6.02万平方米，改造“城中村”15个、老小区146万平方米、老校舍8.8万平方米、老街巷65条。住房公积金新增扩面8万人。残疾人助岗工程和第四轮农村扶贫工程顺利推进。

五、“三创一申”成果丰硕

创成“全国文明城市”。坚持“创建为民、创建惠民、创建靠民、创建育民”理念，扎实推进文明城市创建各项工作，市民文明素养明显提高，城市形象、品质和美誉度大幅提升，扬州以地级市排名江苏第一、全国第三的优异成绩荣获“全国文明城市”称号，宝应安宜镇、仪征真州镇、高邮菱塘乡、邗江建华村、江都横沟村获“全国文明村镇”称号，邗江双桥康乐社区、市国税局(机关)、苏北人民医院获“全国文明单位”称号。

创成“国家森林城市”。按照“规划先行、重点突破、保障有力”的创森思路，高强度组织推动、高标准项目推动，全市新增造林15万亩，市区新增绿化170万平方米，实现了建设森林城市与增加群众收入、整治城乡面貌、提升人居环境的有机结合。

生态市创建和申遗工作取得阶段性成果。全市创建国家生态市通过省级考核验收。江都、邗江分别通过国家考核验收和技术评估。全面实施大运河扬州段遗产点修缮和环境整治、唐子城遗址保护展示、扬州城国家考古遗址公园建设，全市有10处遗产点和6段河道列入大运河申遗预备名单。

六、社会建设成效明显

社会事业全面进步。全市新增15所公办幼儿园，维扬中学新校区和京华梅岭中学建成使用，全市高考本二以上达线率提高7.1个百分点。城乡社区卫生服务机构服务能力全面提升，“15分钟医疗急救圈”建设扎实推进，基本药物制度稳步实施。公共文化服务体系建设加快。市文化馆创成国家一级馆。扬州玉器厂入选首批“国家非物质文化遗产生产性保护基地”。马可·波罗纪念馆等一批文博场馆建成开放。市档案馆改扩建工程开工。创成江苏省体育强市，成功举办一批国际国内体育赛事。人口和计划生育公共服务工作得到加强。台湾事务、侨务、外事和工会、青年、妇女、儿童、科普、慈善、老龄、方志等事业又有新的进步。

社会管理和谐有序。新建社区服务中心18个，社区工作站实现全覆盖。荣获“五五”普法全国先进市称号。完善社会治安防控体系，严厉打击刑事犯罪，社会治安持续稳定。行政复议规范化建设扎实推进。信访积案化解成效明显。应急管理得到加强。食品、药品安全监管扎实有效。全面推广企业安全生产标准化和经济责任保险，实现事故死亡人数和经济损失全面下降。国防动员和后备力量建设积极推进。第六次争创全国“双拥模范城”通过国家验收。市残疾人康复中心投入使用。民族、宗教、民防、仲裁、地震等工作扎实开展。

各项改革协调推进。启动实施事业单位绩效工资改革。基本完成基层医疗卫生机构综合改革，市二院、妇幼保健院等公立医院改革试点积极推进。金融改革扎实有效，建立中小企业融资服务体系，新建农村小额贷款公司19家、村镇银行1家。

七、政府自身建设不断加强

政府服务更加高效。启动实施“四个一工程”，将82个单位公开电话统一整合到12345政府服务热线，市政府数据资源中心和“中国扬州”门户网站群启动建设，覆盖社区、农村的便民服务网络基本建成。行政办事“三集中、三到位”更加优化，市级行政服务窗口按时办结率99%。政府新闻发布工作得到加强。

能力作风不断强化。自觉接受人大及其常委会监督，支持人民政协履行参政议政职能，积极听取各民主党派、工商联和社会各界意见。出台市政府能力作风建设23条实施意见，广泛开展“三走进、三服务”和“三下三联三交”活动。创新完善对经济发展和县（市、区）工作考核。行政权力网上公开运行工作继续深化。积极参加市长与人大代表面对面办理建议活动。全年办理市人大代表建议325件、政协提案461件。

廉政建设得到加强。注重从制度、机制层面推动政府对重大工程、重要事项决策的民主化、公开化、科学化。修订完善建设工程招投标管理办法、市区国有土地出让办法和市规委会议事规则。强化行政效能监察，开展工程建设领域突出问题和小金库、公务用车等专项治理。规范财政预算管理，对1000万元以上政府投资项目实行绩效预评估。

各位代表，过去的一年，我们突出保持经济平稳较快发展，突出保障重大工程和城市建设顺利推进，突出保证民生项目按期落实，突出加强政府自身建设，办成了几件大事，办成了一些实事，实现了“十二五”良好开局。这是全市人民在中共扬州市委的坚强领导下，不畏艰难、同心协力、奋力拼搏的结果，是市人大、市政协积极有效监督和大力支持的结果，是上级部门和社会各界热忱关心帮助的结果。在此，我谨代表扬州市人民政府，向全市人民，向人大代表、政协委员，向驻扬单位和所有关心、支持、参与扬州建设和发展的社会各界人士，表示崇高的敬意和衷心的感谢！

在肯定成绩的同时，我们也清醒地认识到，全市经济发展的综合实力还不够强，转型发展、创新发展任务十分艰巨；城乡居民持续增收难度较大，民生工作与群众期望还有差距；区域发展不平衡问题仍较突出，统筹城乡发展还有大量工作要做；社会建设和管理与社会结构的深刻变化还不相适应，政府自身建设仍需不断加强，等等。对此我们必须高度重视，并采取切实措施认真加以解决。

2012年政府工作任务

2012年是党的十八大召开之年，是全面贯彻落实省、市党代会精神、实施“十二五”规划承上启下的重要一年。政府工作的总体要求是：全面贯彻党的十七届六中全会和中央经济工作会议、省第十二次党代会和全省经济工作会议精神，按照市第六次党代会和市委六届二次全会的部署和要求，坚持“稳中求进、进字当先，好中求快、快字当头”的工作导向，突出“稳增长、快转型、促统筹、惠民生、保稳定、优服务”，进一步转变经济发展方式、保持经济平稳较快增长，进一步增强中心城市功能、推进城乡统筹发展，进一步加强社会建设和管理、促进社会和谐稳定，进一步改善人民生活、提升群众的幸福感和满意度，进一步强化政府自身建设、提高公共服务水平，又好又快地推进“三个扬州”建设，奋力开启基本实现现代化新征程。

2012年全市经济和社会发展的主要预期目标是：地区生产总值增长11%。财政一般预算收入增长20%。固定资产投资增长20%。研发投入占地区生产总值比重达到2.1%。城市居民人均可支配收入增长13%，农民人均纯收入增长15%。居民消费价格指数不高于全省平均水平。城镇登记失业率控制在4%以内。节能减排完成省下达任务。重点做好九项工作：

一、着力夯实经济发展基础，保持经济平稳较快增长

持续强力推进工业重大项目建设。坚持新型工业化第一方略和“项目为王”不动摇，加快在建重大项目建设，上海大众整车形成产能，康博多晶硅、华电燃机热电联产、璨扬光电二期等项目建成投产；仪化PTA、中化国际苯酚丙酮及双酚A、中电电气太阳能电池等一批项目开工建设。全年新开工10亿元以上项目40个，力争40亿元以上重大项目县（市、区）全覆盖。全市新增产值过百亿企业1家、过50亿元企业5家。

进一步促进投资消费协调拉动。持续扩大投资需求、优化投资结构，加快培育新的经济增长点，突出抓好200个市级重点项目建设，全年完成投资1000亿元以上。积极培育消费市场，营造放心消费环境，合理引导群众对文化、旅游、家政等方面的消费预期，推进“万村千乡”、“农超对接”和蔬菜平价店建设。推动住房产业转型升级，加强监测监管，保持房地产市场平稳健康发展。提升城市宜居、宜业品质，全力吸引高素质人才来扬就业、创业、置业。

大力扶持中小企业发展。全面落实扶持中小企业发展的“国九条”、“省十八条”和“扬八条”政策。加大企业信贷投放，确保全年工业贷款增速高于全市贷款增速3个百分点。进一步重视培育本土骨干企业，强化金融、技术、人才等支持服务，推动和扶持企业加快技术改造、产品创新和管理升级，全市亿元、10亿元以上企业分别达1300家、120家。

壮大发展建筑业。进一步提升建筑业优势产业和富民产业地位，专项制定实施扶持政策，打响建筑强市品牌。加快实施建筑业“十百千万”行动计划，支持骨干企业开拓市场，推动建筑产业向高端延伸。全年建筑业总产值突破2000亿元。

二、着力调整产业结构，加快转变经济发展方式

大力实施人才战略。坚持“人才为纲”，把人才作为经济和城市发展的第一资源，把人才战略放在特别优先发展的位置。按照“大幅度扩充总量、大力度调整流向”的总目标，系统研究制定并启动实施人才“1111工程”。特别是要认真研究企业人才需求和人才自身发展需要，以改善人才生活条件尤其是解决住房问题为关键，制定实施最专业、最优惠的人才招引政策。同时，大幅度增加政府人才专项投入，强化人才服务，为人才“引得来、用得好、留得住”创造最佳环境。

加快发展现代服务业。围绕服务业“比重提升、水平提升、价值提升、竞争力提升”的总要求和“十二五”末“两个48%”的目标，全力实施九大重点产业提速发展行动计划。重点围绕世界500强、国内50强服务业企业引进一批重大项目。加快宜游城市和旅游产业强市建设。加快软件与信息服务、文化创意、金融业等8大集聚区建设，创成2个省级现代服务业发展示范区。启动软件服务业“512”培育计划、物流业“345”行动计划。全年服务业增加值占地区生产总值比重达到40%以上。

快速发展新兴产业。引导和支持“三新一网一书”新兴产业企业加强对核心技术、关键环节的攻关与转化，不断提升核心竞争力。利用“十城万盏”、“三网融合”、“电子书进课堂”等政策和机遇，强化国内外市场开拓，扶持培育一批龙头企业。对前景看好、眼前困难的新兴产业特别是产业链重要环节中的中小企业适时制定实施扶持政策。全市“三新一网一书”产业产值2200亿元。认真研究国家鼓励实体经济、发展新兴产业政策，密切跟踪研究世界新兴产业发展趋势，继续培育新的新兴产业。

加速提升科技创新能力。全面实施“千企创新升级”计划，新增国家高新技术企业50家以上，新认定市级创新型（试点）企业140家，高新技术产业实现产值3500亿元。突出企业创新主体地位，推动规模以上工业企业研发中心全覆盖、研发经费占销售收入1.2%以上。狠抓创新载体建设，加快提升省级、国家级开发园区发展质量，打造扬州科技园、汽车科技城等一批创新发展载体，推动邗江经济开发区创建省高新技术产业开发区。加强与重点工科院校和科研院所合作，新建校企联盟100家以上，签约产学研合作项目300项以上。加快“智慧城市”建设，推进信息化与工业化深度融合，力争创成国家创新型试点城市。推动发展科技金融，打造覆盖不同创新阶段的金融服务支撑体系。深入实施专利、品牌、标准、上市“四大战略”。

坚决抓好节能减排。抓好重点领域、重点行业的节能减排，加快淘汰落后产能。加大节能环保技术、循环利用技术、可再生资源技术以及新型节能建材等新技术、新产品的推广应用，全年创建节能示范企业28家，实施循环经济试点项目30个。加快推进环保科技产业园发展。

三、着力抓好“三农”工作，提高城乡统筹发展水平

全面实施农业现代化工程。稳定粮食生产，新增高标准农田15万亩以上，打造50个万亩高产示范方。加快高效设施农业规模化，提升建设农业亮点工程，引进项目600个以上。推动农业科技创新，全面建成“五有”乡镇农技推广综合服务中心，大力推进农业机械化及秸秆还田综合利用。进一步提升农业产业化水平，培育100个市级以上“五好”示范社，市级以上农业龙头企业销售增长15％以上。认真实施“米袋子”、“菜篮子”工程，加强源头控制和检验检测，新增“菜篮子”基地2500亩、“三品”品牌120个以上。

大力推进水利现代化建设。推进淮河入江水道整治工程，完成投资5亿元。基本完成南水北调一期工程建设任务，完成仪扬河、白塔河等中小河流治理。建成乌塔沟分洪道。推进长江嘶马弯道杨湾段应急护岸工程和新砂滩、扇子圩河口水利血防工程。继续疏浚县乡河道、村庄河塘。更新改造老化失修的小型泵站300座。

加快提升城镇承载服务能力。提升建设县城镇，进一步增强辐射带动力。做强11个重点中心镇，加快基础设施和公共服务体系建设，推进“强镇扩权”改革。提高一般镇规划建设水平，增强配套服务功能。加快推进“三集中”，抓好万顷良田建设工程、城乡建设用地增减挂钩试点，新建50个农民集中居住区。

全面开展“美好城乡建设行动”。对规划布点村庄按照“六整治、六提升”的要求进行环境综合整治，创成省三星级“康居乡村”20个以上。对非规划布点村庄按照“三整治、一保障”的要求进行环境卫生整治，20%的非规划布点村庄达到省“环境整洁村庄”标准。突出整治重点，率先推进高速公路沿线、城镇主要出入口及“五路一带”沿线村庄环境整治。进一步优化完善村庄布局规划和建设规划。巩固提升农村“四位一体”长效管护建设成果，拓展管护范围，提高管护水平。

扎实推进村级组织“四有一责”建设。着力提升村级组织发展集体经济、完善公共服务、组织农民合作、强化民主管理的能力。全市集体经营性收入低于5万元的208个薄弱村，全部建成创收20万元以上的物业、产业项目载体。

四、着力深化改革开放，不断激发发展活力

有力有效推进经济国际化。深入实施“530”招商行动计划，推动开展“名人名企名城行”活动。办好“烟花三月”国际经贸旅游节、世界运河名城博览会暨世界运河大会。调整优化出口结构，进一步扩大外贸出口。支持企业参与境外项目投资开发，加快“走出去”步伐。进一步推进园区建设，完善园区功能、加快产业发展，不断提升园区竞争力。

放手放开发展民营经济。鼓励非公有制经济进入金融服务、公用事业、高新产业和基础设施等领域，支持企业跨行业、跨地区兼并重组。加大特色产业基地培育力度，形成专业特色乡镇板块集群40个。抓好节庆招商，重视以商引商，招引10亿元以上民资项目15个以上，新增开工亿元以上民资项目100个。全年新增民营企业注册资本金实际到资增长20%，注册亿元以上有限公司增长30%，新增个体工商户2.5万户。

继续深化重点领域和关键环节改革。分类推进事业单位改革。积极推进文化体制改革，促进文化创新发展。继续深化医药卫生体制改革，加快公立医院改革步伐。扎实做好统筹城乡发展综合配套改革。进一步抓好农村土地承包经营权确权、登记、发证试点，有序推进村经济合作社建设。大力发展政策性农业保险。积极推进其他领域各项改革。

五、着力推进融合发展，做大做强中心城市

加快推进宁镇扬同城化。启动宁扬城际轨道前期工作。有序推进医保、教育等同城化，研究细化电信、旅游“市民同城待遇”的具体举措。协同开展水利、环保、警务、农产品质量监管等工作。

加强重大基础设施建设。确保扬州泰州机场按期通航。继续推进宁启铁路复线及电气化改造、扬州港“一港三区”、扬州西500千伏输变电工程等项目建设。建成沪陕高速江六段。加快推进扬宿高速及237省道扬州段、333省道京沪高速以西段等国省干线公路建设。抓紧京沪高速扩容、沪陕高速江广段等项目前期工作。紧密跟踪推进连淮扬镇铁路和长江公铁大桥项目。

整合推进市区一体化发展。根据区划调整后的新情况，制定实施“市区融合发展三年行动计划”。修编完善城市总体规划和土地利用规划，优化城市空间、产业、重大基础设施和公共服务设施布局。认真做好江都与广陵接合部的建设发展规划。加强三区之间、乡镇（街道）之间、园区之间资源和产业整合，推进集聚集约发展。加强基础设施对接，适时启动新万福路等项目建设，逐步加密主城区与江都区公交线网。推进民生保障一体化，新的民生项目实行“同城同步同标”，原有民生项目按照“就高不就低”的原则“一年厘清、三年补平”。

提升城市功能品位。全力推进瘦西湖地下隧道工程建设。文昌路东延段建成通车。扬菱路改造全面完成。加快实施328国道城区连接线。整治史可法北路、兴城东路等城市道路，大力推进“五路一环一河”环境提升。通过“外防、内排、活水、治淮”相结合，实施城市系统治水。启动赵庄垃圾填埋场扩容和垃圾焚烧发电厂二期建设。筹建六圩污水处理厂三期。巩固森林城市创建成果，实施新一轮“绿杨城郭新扬州”三年行动计划，全市新增造林8万亩，市区新增绿化100万平方米。统筹推进广陵新城建设，启动建设金融集聚区。加快推进新城西区拓展区和扬州经济技术开发区临港新城建设。完善建设“双东”历史街区，启动南河下历史街区整治。提高城市精细管理水平，不断改善市容环境。

全力争创国家生态市。大力实施“碧水、蓝天、宁静”工程，重点抓好大气、水体、重金属和农业面源污染防治，加快“十二五”淮河流域和长江流域水污染防治步伐。进一步改善环境质量，确保全市饮用水源水质达标率100%，市区空气质量优良天数320天以上。

六、着力建设文化强市，推动社会事业全面发展

推动文化大发展大繁荣。大力实施文化建设“十大工程”，突出抓好公共文化服务体系建设，新建农村文化广场200个，推动公共文化设施免费开放。继续推进文化博览城建设，加快两淮盐政史料陈列馆、文汇阁等一批文博场馆建设，实施名城解读50处。巩固文明城市创建成果，认真研究“扬州好人”蔚然成风的典型效应和形成机理，针对不同群体选树不同典型，努力形成覆盖所有人群、富有扬州特色的城市荣誉体系。深入推进学习型城市建设，举办“4·23”世界读书日活动。加快发展文化产业，重点推进国家数字出版（扬州）园区、人民大学文化科技园、凤凰文化广场、扬州现代传媒广场项目建设。扶持原创性和地域性文艺作品创作，抓好电视剧《大清盐商》、扬剧《青春树》等作品创作与加工。着眼于迎接扬州2500周年城庆，启动编辑《扬州文库》。

提升发展教育事业。加快学前教育普惠优质发展，80%乡镇建有一所优质公办幼儿园。推进义务教育优质均衡发展，完成中小学校舍安全工程年度任务。全市新招宏志班20个。突出抓好普通高中教育，全市80%的学生在省三星级以上普通高中就读。加强职业教育。支持扬州大学建设，扶持市属高校发展。

加快发展医疗卫生事业。进一步完善“15分钟医疗急救圈”，加快建设区域卫生信息平台，城乡社区卫生服务中心（卫生院）标准化率95%以上。实施41项免费基本公共服务项目，居民电子健康档案建档率60%以上。提高新农合人均筹资标准、报销比例和最高支付限额。实现村卫生室基本药物零差率销售。启动市中心血站和苏北医院急诊中心建设，搬迁市惠民医院。

进一步推动申遗工作。认真履行牵头城市职责，协助做好大运河联合申遗工作。实施遗产点修缮和环境整治，申报一批重点项目，争取更多遗产点列入大运河申遗文本。完成扬州地方项目申遗文本和规划编制并进入国家申遗预备名单。

统筹推进其他社会事业。进一步组织实施全民健身“三边”工程，加快体育公园体育场和体校二期工程建设，办好第七届鉴真国际半程马拉松等赛事。继续抓好人口问题综合治理和计生优质服务。推进残疾人事业、老龄事业和慈善事业。积极做好外事、侨务工作。完成《扬州市志（1988～2005）》总纂。全面实施《全民科学素质行动计划纲要》。做好新疆新源县等对口支援工作。积极抓好民兵预备役建设，全面普及国防教育，巩固“双拥模范城”创建成果。完善公共气象服务体系建设，提高防灾减灾能力。

七、着力保障和改善民生，不断提升人民群众幸福感

全面实施居民收入倍增计划。把充分就业、勤劳创业、发展实业作为增收的主渠道，落实就业创业“四项行动计划”，全市新增城镇就业5万人、农村劳动力转移1.4万人，创业培训4000人，新建创业孵化基地10个。市区新增政府购岗500个，城镇零就业家庭保持动态清零。建立和谐劳资关系，深入推进企业职工工资集体协商制度。

完善提升社会保障。全市企业职工基本养老保险新增参保3.2万人，参保率达98%以上。新农保参保率、补贴发放率均达98%以上。职工基本医疗保险、城镇居民基本医疗保险和新型农村合作医疗参保率均达98%以上。工伤保险新增参保3.2万人。邗江、广陵、扬州经济技术开发区被征地农民和失地农民转参企业职工养老保险覆盖率95%以上。建立完善社会救助、优抚标准自然增长机制，稳步提高城乡低保和农村“五保”标准，落实低收入群体物价上涨动态补贴机制。

全力办好民生实事。实现区域供水全覆盖。加强民生价格监控。加强食品、药品安全监管。继续推进保障性住房建设，提高保障对象收入等限定标准，市区新建廉租房280套、经济适用房1400套、公共租赁房4800套（间）。继续推进“八老”改造，市区整治老小区155万平方米、公房解危6万平方米、改造“城中村”15个。新建老城区、主要景点周边和主干道沿线公共厕所10座。推进公交优先，优化调整公交线路12条。科学布局市区文体设施。大力推进养老服务体系建设，全市新建120个社区（村）居家养老服务中心。深入开展农村扶贫，全市75%的低收入农户年人均纯收入达到3600元以上。

八、着力加强社会管理，促进社会和谐稳定

深化和谐社区建设。全面深化完善“四位一体”社区治理模式，建立社区管理综合信息系统，支持社会组织和社区志愿者参与社区管理。健全公共服务、志愿服务、有偿服务相衔接的社区服务体系，不断增强社区服务功能。大力开展社区创先争优活动，完善设施、美化环境，加快提升社区建设水平。

畅通群众利益诉求渠道。认真办好12345政府服务热线，回复率和满意率分别达90%和70%以上。完善信访工作长效机制，高度重视并切实解决好群众关心的征地、拆迁等热点难点问题。加大法律援助力度，引导群众依法合理表达利益诉求。密切关注社会动态和网络舆情，健全完善社情民意回应机制。

强化安全生产。严格落实安全生产责任制，完善政府监管、企业主体和属地管理的安全生产责任体系。加大执法监管力度，继续突出抓好重大安全隐患的排查和整治，加强对道路交通、校车、消防、建筑施工和危险化学品等重点行业领域的安全监管，坚决打击安全生产违法行为。

维护社会稳定。认真实施“六五”普法规划。推进社会管理综合治理工作，不断完善社会治安打防控体系。加强社会稳定风险评估，建立和完善矛盾纠纷调处化解综合平台。切实抓好未成年人思想道德教育。加快推进社会信用体系建设。高度重视民族、宗教工作。加强网络管理。进一步健全应急体系，提高应对处置重大突发事件的能力。

九、着力建设服务型政府，努力提高服务实效

注重学习调研。自觉加强对新知识、新技能、新业务及国内外先进经验的学习钻研，以学习开阔视野胸襟、创新思路办法、转变能力作风。继续加强公务员培训，不断提升工作的专业化水平。深入开展“三下三联三交”活动，对涉及民生的规划、交通、城市管理等重要事项，普遍建立公示和征求市民意见制度，切实问政于民、问计于民。

提升服务效能。牢记责任就是使命，加强机关效能建设，着力治庸、治懒、治散。敢于突破、敢于担当，以改革创新的办法化解发展中碰到的矛盾和困难。围绕企业所急所需，广泛开展“进厂入企”和“一企一策、特事特办”等服务活动，与企业共渡难关。密切关注国家和省财政投资项目动态，积极采取多渠道融资、向上争取、整合资源等方式，保证重大工程建设资金需求。

强化依法行政。自觉接受人大及其常委会的法律监督、工作监督和政协民主监督，高度重视司法监督、舆论监督和人民群众监督，主动听取各民主党派、工商联、无党派人士和人民团体意见。不断提高人大代表议案、建议和政协提案办理的解决率。继续深化行政审批制度改革。全面开展行政指导和行政调解工作，强化行政复议规范化建设。进一步完善重大行政决策制度，加大政府信息公开力度，提升行政权力网上运行质量。办好政府新闻发布和“寄语市长”，着力打造公开透明的政府。

加强廉政建设。认真落实廉政建设责任制，健全惩治和预防腐败体系，深入开展反腐败斗争。深化权力内控体系建设。加大对财政资金、重大投资项目和民生事项的审计力度，加强对重大工程建设的监督检查，健全完善责任追究制度。加强公务员理想信念和廉洁自律教育，大力弘扬为民、务实、清廉作风，努力建设一支为人民服务、对人民负责、受人民监督、让人民满意的公务员队伍。

各位代表，全面完成本届政府的各项目标任务，向全市人民交上一份满意的答卷，我们深感责任重大，使命光荣。让我们在中共扬州市委的领导下，认清形势、团结拼搏，抓住机遇、开拓创新，咬定目标、奋发前行，为全面提升“三个扬州”建设水平，不断开创经济社会发展新局面，以优异的成绩迎接党的十八大胜利召开而努力奋斗！
